# Integrase Defective Lentiviral Vector Promoter Impacts Transgene Expression in Target Cells and Magnitude of Vector-Induced Immune Responses

**DOI:** 10.3390/v15112255

**Published:** 2023-11-14

**Authors:** Sneha Mahesh, Jenny Li, Tatianna Travieso, Danai Psaradelli, Donatella Negri, Mary Klotman, Andrea Cara, Maria Blasi

**Affiliations:** 1 Department of Medicine, Division of Infectious Diseases, Duke University School of Medicine, Durham, NC 27710, USA; smahesh7@jh.edu (S.M.); jenny.j.li@duke.edu (J.L.); tatianna.travieso@colostate.edu (T.T.); danai.psaradelli@duke.edu (D.P.); donatella.negri@duke.edu (D.N.); mary.klotman@duke.edu (M.K.); andrea.cara1@duke.edu (A.C.); 2 Duke Human Vaccine Institute, Duke University School of Medicine, Durham, NC 27710, USA; 3 Department of Infectious Diseases, Istituto Superiore di Sanità, 00161 Rome, Italy; 4 National Center for Global Health, Istituto Superiore di Sanità, 00161 Rome, Italy

**Keywords:** lentiviral vectors, dendritic cells, muscle cells, antigen expression, vector promoter, immune responses

## Abstract

Integrase defective lentiviral vectors (IDLVs) are a promising vaccine delivery platform given their ability to induce high magnitude and durable antigen-specific immune responses. IDLVs based on the simian immunodeficiency virus (SIV) are significantly more efficient at transducing human and simian dendritic cells (DCs) compared to HIV-based vectors, resulting in a higher expansion of antigen-specific CD8+ T cells. Additionally, IDLV persistence and continuous antigen expression in muscle cells at the injection site contributes to the durability of the vaccine-induced immune responses. Here, to further optimize transgene expression levels in both DCs and muscle cells, we generated ten novel lentiviral vectors (LVs) expressing green fluorescent protein (GFP) under different hybrid promoters. Our data show that three of the tested hybrid promoters resulted in the highest transgene expression levels in mouse DCs, monkey DCs and monkey muscle cells. We then used the three LVs with the highest in vitro transgene expression levels to immunize BALB/c mice and observed high magnitude T cell responses at 3 months post-prime. Our study demonstrates that the choice of the vector promoter influences antigen expression levels in target cells and the ensuing magnitude of T cell responses in vivo.

## 1. Introduction

Lentiviral vectors (LVs) are efficient tools for expressing genes of interest in diverse cell types in vitro and in vivo. Lentiviral vectors offer several advantages as gene therapy tools and vaccine platforms, including an ability to transduce both dividing and non-dividing cells, a relatively large cargo capacity, a variety of alternative envelope proteins for virion pseudotyping, an absence of pre-existing host immunity and an ability to maintain a persistent gene expression [1,2,3,4,5]. 

While lentiviral vectors show a lower risk of genotoxicity compared to other retroviral vectors, integrase defective lentiviral vectors (IDLVs) have been developed to further avoid risks associated with insertional mutagenesis, especially when using this platform as a vaccine. These viral vectors have been mutated in long terminal repeats (LTRs), the packaging signal and the integrase gene to render them self-inactivating, non-replicating and non-integrating. IDLV persists in the target cell in an episomal form and can express the encoded gene for the lifetime of the cell. Our group developed both HIV- and SIV-based IDLVs to deliver a broad range of proteins for the induction of durable antigen-specific immune responses in both mice and non-human primates (NHPs) [6,7,8,9,10,11,12,13]. IDLVs under development for vaccine delivery can offer significant advantages in addressing the limitations shown by other vaccine platforms. In addition to their ability to efficiently transduce both dividing and non-dividing cells, IDLVs stimulate a potent and durable antigen-specific immune response and are not limited by pre-existing anti-vector immunity [6,14,15]. In extensive safety studies by our group and others, no replication competent lentiviruses (RCLs) were detected after IDLV immunization [9,12,13]. We have shown that a single intramuscular (IM) injection with an SIV-based IDLV expressing an HIV-1 Env under the ubiquitous cytomegalovirus (CMV) promoter induced broad and sustained antigen-specific immune responses for up to 1 year post-injection in NHPs [9] and stimulated antibody affinity maturation [12]. Additionally, immunization of NHPs with IDLV induced higher-magnitude and more durable binding and neutralizing Ab responses compared to the other vaccine regimens, including just protein and DNA +/− protein [13]. The long-term immunity induced by IDLV may be ascribed to the persistence of IDLV in vivo. Indeed, the retro-transcribed episomal vector DNA can still be detected in the muscle of immunized NHPs at 6 months post-injection [12] and, in mice, transgene expression was detected up to 3 months post-injection in muscle tissue [12,16], albeit at lower levels compared to 3 days post-injection, suggesting that IDLV can provide persistent transgene expression at the injection site. The induction of durable protective immune responses is a desirable feature for any vaccine, eliminating the need to perform repeated immunizations every few months. Given the critical role of dendritic cells (DCs) in the induction of immune responses and of muscle cells in the maintenance of an antigen reservoir following IDLV injection, we generated and tested ten novel SIV-based lentiviral vector constructs expressing green fluorescent protein (GFP) as a model antigen to readily assess expression and immunogenicity under different hybrid promoters to further optimize transgene expression levels in both DCs and muscle cells. Transgene expression levels from the novel LVs were compared to those of LVs expressing GFP under the commonly used CMV or CMV early enhancer/chicken β actin (CAG) promoters in both mouse- and rhesus-macaque-derived DCs as well as in monkey muscle cells. To evaluate whether the high transgene expression levels observed in in vitro-transduced DCs and muscle cells translated to a high magnitude of immune responses in vivo, we used three of the novel IDLVs expressing GFP to immunize BALB/c mice and measured T cell responses 3 months post-immunization. Our results show that two of our novel SIV-based IDLVs that achieved higher transgene expression levels in in vitro-transduced DCs and muscle cells also induced a high magnitude of T cell responses compared to the IDLV expressing the transgene under the commonly used CMV promoter. 

## 2. Materials and Methods

### 2.1. Plasmid Construction and Lentiviral Vector Production 

The hybrid promoters (PrDrive1–10) shown in Table 1 were cloned into an SIV-based lentiviral transfer vector expressing GFP [8]. For vector production, the newly generated plasmids were co-transfected together with either the integrase competent packaging plasmid (pAd-SIV-3+) for the in vitro studies, or the integrase defective packaging plasmid (pAd-SIV-D64V) for the in vivo studies and the pseudotyping plasmid expressing the vesicular stomatitis virus glycoprotein (VSV-G) of the Indiana serotype (pVSV.G_IND_) into 293T Lenti-X cells as previously described [12]. Briefly, the human epithelium kidney 293T Lenti-X cells (Clontech Laboratories, Mountain View, CA, USA) were cultured in Dulbecco’s Modified Eagle Medium (DMEM) (Thermo Fisher Scientific, Waltham, MA, USA, Cat# 11965092) containing 10% fetal bovine serum (FBS) (GE Healthcare Life Sciences, HyClone Laboratories, South Logan, UT, USA) and 100 units/mL of penicillin–streptomycin (PS) (Thermo Fisher Scientific, Waltham, MA, USA, Cat# 15140-122). A total of 3.5 × 10^6^ 293T Lenti-X cells were seeded on 100 mm diameter Petri dishes (Corning, Corning, NY, USA, Cat# 430167) and transfected with 8 µg per plate of a plasmid mixture containing transfer vector, packaging plasmid and VSV.G plasmid in a 2:4:2 ratio, using the JetPrime transfection kit (Polyplus Transfection, Illkirch, France, Cat# 55-134) following the manufacturer’s recommendations. At 48 and 72 h post-transfection, culture supernatants were collected, and cellular debris was removed by low-speed centrifugation and filtration through a 0.45 μm pore size filter unit (Nalgene, Rochester, NY, USA, Cat# 125-0045). Filtered supernatants were concentrated by ultracentrifugation for 2 h at 23,000 RPM on a 20% sucrose cushion. Vector particles were resuspended in 1× phosphate-buffered saline (PBS) and stored at −80 °C until further use. LV and IDLV stocks were validated and titered by flow cytometry (BD Biosciences, Franklin Lakes, NJ, USA) and by a colorimetric reverse transcriptase (RT) assay (Roche, Basel, Switzerland, Cat# 11468120910) as previously described [9,17]. Briefly, 5 × 10^4^ 293T Lenti-X cells were seeded on 12 well plates and transduced the following day with serial dilutions of either LV-GFP or IDLV-GFP stock. At 72 h post-transduction, the percentage of GFP positive cells was assessed by flow-cytometry and the vector titers were calculated using the following formula: transducing unit (TU) = dilution factor × number of cells per well × percentage of GFP positive cells (expressed in decimals). 

### 2.2. LV Transduction of 293T Lenti-X Cells

A total of 50,000 293T Lenti-X cells were plated in each well of a 12-well plate (Coning, Corning, NY, USA, Cat# 353043) with DMEM (Thermo Fisher Scientific, Waltham, MA, USA, Cat# 11965092) supplemented with 10% FBS (GE Healthcare Life Sciences, HyClone Laboratories, South Logan, UT, USA) and 100 units per mL PenStrep (Thermo Fisher Scientific, Waltham, MA, USA, Cat# 15140-122). The following day, cells were incubated overnight with the different lentiviral vectors at a multiplicity of infection (MOI) of 1 in triplicate. GFP expression and mean fluorescence intensity (MFI) were analyzed by flow cytometry (BD Biosciences, Franklin Lakes, NJ, USA) using the FlowJo software (FlowJo LLC V10, Ashland, OR, USA) at 2, 7, 9, 12 and 14 days post-transduction (Supplementary Figure S1).

### 2.3. LV Transduction of Mouse-Bone-Marrow-Derived Dendritic Cells

Bone-marrow-derived dendritic cells (BMDC) were derived from WT C57BL/6 by culturing the bone marrow progenitor cells in RPMI 1640 medium (Thermo Fisher Scientific, Waltham, MA, USA, Cat# 11875093) supplemented with 10% FBS (GE Healthcare Life Sciences, HyClone Laboratories, South Logan, UT, USA), 100 units per ml PenStrep (Thermo Fisher Scientific, Waltham, MA, USA, Cat# 15140-122) (cRPMI), 20 ng/mL of GM-CSF (Miltenyi Biotech, Bergisch Gladbach, North Rhine-Westphalia, Germany, Cat# 130-094-043) and 40 ng/mL of Flt3-ligand (Miltenyi Biotech, Cat# 130-094-038). The cells were kept in culture for 6 days in a 100 mm diameter Petri dish (Corning, Corning, NY, USA Cat# 430167) before differentiated BMDCs were harvested. Isolation of BMDCs was verified by flow cytometry (BD Biosciences, Franklin Lakes, NJ, USA) following incubation of the cells with a FITC-labeled CD11c antibody (Biolegend, San Diego, CA, USA, Cat# 117301). A total of 100,000 BMDC were plated in each well of a 24-well plate (Corning, Cat #353047) and transduced with the lentiviral vectors at an MOI of 2 (based on 293T Lenti-X titers). cRPMI supplemented with the previously mentioned cytokines was added to the cells the following morning and every 2 days for the remainder of the experiment. GFP expression was analyzed by flow cytometry (BD Biosciences) at 3 and 7 days post-transduction (Supplementary Figure S2).

### 2.4. LV Transduction of Monkey-Monocyte-Derived Dendritic Cells

CD14+ monocytes were isolated from rhesus macaque peripheral blood mononuclear cells (PBMC) using magnetic separation of non-human primate CD14 microbeads (Miltenyi Biotech, Bergisch Gladbach, North Rhine-Westphalia, Germany, Cat# 130-091-097; MACS cell separation columns and separator, Cat# 130-042-401 and 130-042-302). Monocytes were differentiated into DCs using cRPMI containing 100 ng/mL human GM-CSF (Peprotech, Cranbury, NJ, USA, Cat# 300-25) and 100 ng/mL human IL-4 (Peprotech, Cranbury, NJ, USA, Cat# 200-04) on a 100 mm diameter plate (Corning, Corning, NY, USA, Cat# 430167). cRPMI supplemented with the previously mentioned cytokines was added to the cells 3 days post-isolation and every 2 days for the remainder of the experiment. At 7 days post-isolation, 14,000 DCs were plated in each well of a 24-well plate (Corning, Cat# 353047) and transduced with each of the LVs listed in Table 1 at an MOI of 4 (based on 293T Lenti-X titers) in duplicate. Half of the medium (cRPMI+cytokines) was changed the morning after transduction. GFP expression was analyzed by flow cytometry (BD Biosciences, Franklin Lakes, NJ, USA) 3 and 7 days post-transduction (Supplementary Figure S3).

### 2.5. LV Transduction of Human-Monocyte-Derived Dendritic Cells

CD14+ monocytes were isolated from human peripheral blood mononuclear cells (PBMC) using magnetic separation of human CD14 microbeads (Miltenyi Biotech, Bergisch Gladbach, North Rhine-Westphalia, Germany, Cat# 130-050-201; MACS cell separation columns and separator, Cat# 130-042-401 and 130-042-302). Healthy donor PBMCs were purchased from the Gulf Coast Regional Blood Center (Houston, TX, USA). Monocytes were differentiated into DCs using cRPMI containing 100 ng/mL human GM-CSF (Peprotech, Cranbury, NJ, USA, Cat# 300-25) and 100 ng/mL human IL-4 (Peprotech, Cranbury, NJ, USA, Cat# 200-04) on a 100 mm diameter plate (Corning, Corning, NY, USA, Cat# 430167). cRPMI supplemented with the previously mentioned cytokines was added to the cells 3 days post-isolation and every 2 days for the remainder of the experiment. Differentiation into DCs was verified by flow cytometry (BD Biosciences, Franklin Lakes, NJ, USA) following incubation of the cells with a PE-labeled mouse anti-human CD1a antibody (BD Biosciences, Franklin Lakes, NJ, USA Cat# 561754). At 7 days post-isolation, 14,000 DCs were plated in each well of a 24-well plate (Corning, Corning, NY, USA, Cat# 353047) and transduced with each of the LVs listed in Table 1 at an MOI of 4 (based on 293T Lenti-X titers) in duplicate. Half of the medium (cRPMI+cytokines) was changed the morning after transduction. GFP expression was analyzed by flow cytometry (BD Biosciences, Franklin Lakes, NJ, USA) 3 and 7 days post-transduction (Supplementary Figure S4).

### 2.6. LV Transduction of Monkey Skeletal Muscle Cells

Cynomologus macaque skeletal muscle cells (Cell Biologics, Chicago, IL, USA, Cat# MK-6167) were maintained in a complete smooth muscle-cell medium (Cell Biologics, Cat# M2268). A total of 25,000 cells were seeded in each well of a 12-well plate (Coning, Corning, NY, USA, Cat# 353043) previously coated with 0.1% gelatin (Sigma-Aldrich, St. Louis, MO, USA, Cat# G1393). Cells were then transduced with each of the LVs listed in Table 1 at an MOI of 0.5 (based on 293T Lenti-X titers) in duplicate. The medium was changed the morning following transduction. GFP expression was analyzed by flow cytometry (BD Biosciences, Franklin Lakes, NJ, USA) and fluorescence microscopy (ZEISS Axiovert A1 inverted fluorescence microscope) 3 and 7 days post-transduction (Supplementary Figure S5).

### 2.7. Mouse Immunization Study

Five groups of 5 Balb/C mice (2 male, 3 female) were immunized once intramuscularly with 50 ng of RT equivalent (corresponding to 5 × 10^6^ transducing units (TU)) of IDLVs expressing GFP under promoters PrDrive2, 6 and 8 and CMV (Groups 1–4) or saline (Group 5). Twelve weeks post-immunization, the mice were euthanized and spleens were recovered for analysis of cellular immune responses. Splenocytes were cryopreserved and stored in liquid nitrogen until assayed.

### 2.8. IFN-γ ELISpot Assay

For the assay, 96-well plates were coated overnight with 5 µg/mL of purified anti-mouse interferon-γ (IFN-γ) (BS Biosciences, Franklin Lakes, NJ, USA, Cat# 551083) diluted in D-PBS. Plates were washed once with cRPMI, and then blocked for 2 h at room temperature using 200 µL per well of cRPMI. After blocking, the plates were aspirated and 200K splenocytes per well were added and stimulated with either GFP peptide (H-2d-restricted 9-mer peptide HYLSTQSAL, 4 µg/mL) (ProImmune UK, Oxford, UK, Cat#P198-0A) or the antigen-independent mitogen concanavalin A (10 µg/mL) (Sigma-Aldrich, Darmstadt, Germany, Cat# C0412-5MG) in cRPMI. Plates were incubated overnight. The following day, plates were washed 2 times with deionized water and 3 times with PBS containing 0.05% of Tween-20 (Sigma-Aldrich, Darmstadt, Germany, Cat# P1379) and incubated with 2.5 µg/mL biotinylated anti-mouse IFN-γ (BS Biosciences, Franklin Lakes, NJ, USA, Cat# 551083) for 2 h at room temperature. Subsequently, plates were washed 3 times with PBS containing 0.05% Tween-20 and incubated with streptavidin-HRP 100X for 1 h at room temperature. Following incubation, cells were washed 4 times with PBS containing 0.05% Tween-20 and 2 times with PBS, and the substrate solution (NovaRed, Vector Laboratories, Newark, CA, USA, Cat#101098-448) was allowed to develop for 5 min. After spot development, plates were washed with deionized water, air-dried and read using the ImmunoSpot Capture software version 7.0.26.0 (Cellular Technology Limited, Shaker Heights, Cleveland, OH, USA). A response was considered positive when there was at least a 2-fold increase in the number of spots over medium-treated wells (background), with a minimum threshold of 50 SFCs per million splenocytes in the stimulated wells.

## 3. Results

### 3.1. Vector Promoters Impact Transgene Expression Levels in Different Cell Types

To determine the impact of different promoters on transgene expression levels in various cell types, we cloned the ten hybrid promoters shown in Table 1 into an SIV-based transfer vector plasmid encoding for GFP. These hybrid promoters are made of enhancers, core promoters, and 5′ untranslated regions (UTRs) of various origins (Table 1). The newly generated plasmids were then used to generate integrase competent lentiviral vector (LV) particles for subsequent transduction experiments in various cell types.

**Figure 1:**
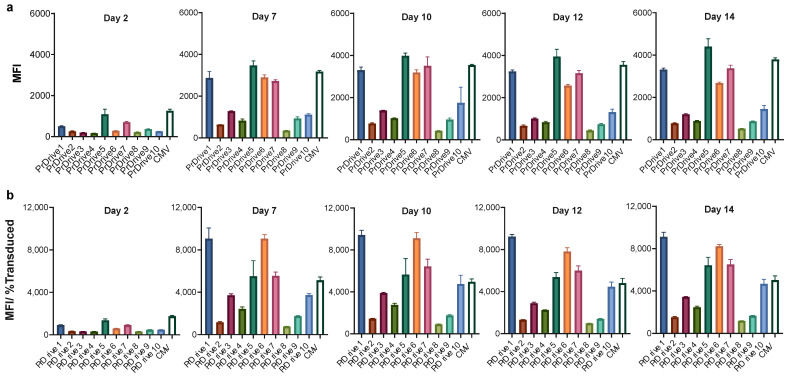
Impact of vector promoter on transgene expression in 293T Lenti-X cells over time. 293T Lenti-X cells were transduced with a multiplicity of infection (MOI) of 1 using the LVs expressing GFP under the indicated promoters. (**a**) Results of time-course flow cytometric analyses performed at the indicated time points to compare the mean fluorescence intensity (MFI) among the different LVs. (**b**) MFI normalized by the percentage of transduced cells expressing GFP, to account for differences in transduction efficiency. Histograms show the means of three replicates. Error bars indicate standard error mean (S.E.M).

We first assessed the transgene expression levels achieved by each of the newly generated LVs on 293T-Lenti-X cells. LVs expressing GFP under the commonly used CMV or CAG promoters were used as controls. As shown in Figure 1a,b, LVs expressing GFP under promoters PrDrive1, 5 and 7 together with the CMV promoter had the highest transgene expression levels (measured as mean fluorescence intensity, MFI) at 2 days post-transduction. At 7 days post-transduction, the four previous LVs along with the LV expressing GFP under the PrDrive6 promoter had MFIs that were twice as high as those of the rest of the LVs. The MFIs for all vectors increased from Day 2 to Day 7 post-transduction and then remained at similar levels from Day 7 to 14, suggesting that, in these cells, peak GFP expression occurs at 7 days post-transduction.

**Figure 2:**
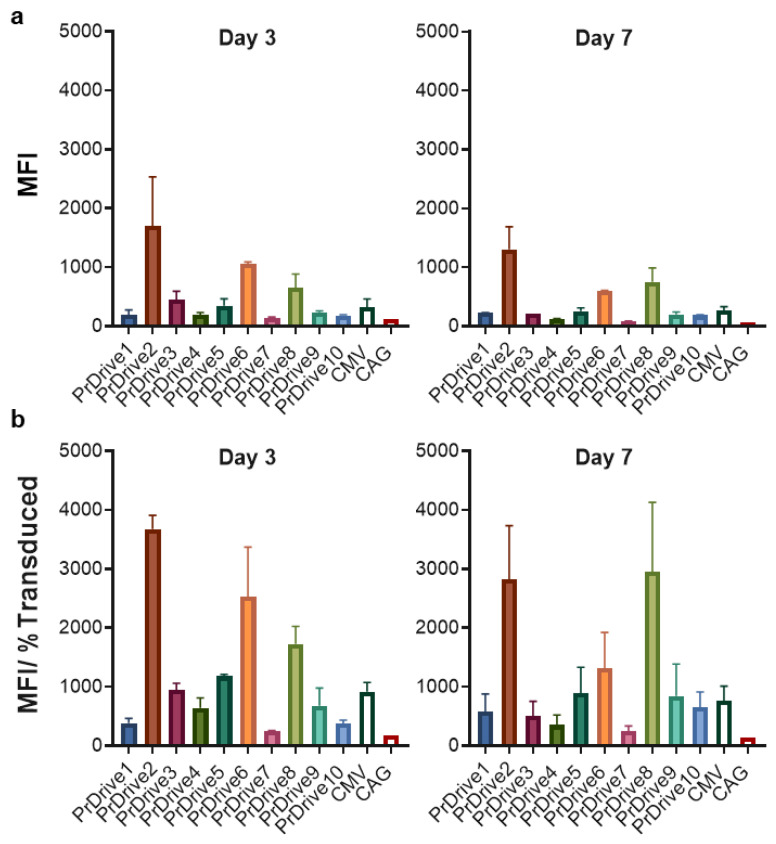
Impact of vector promoter on transgene expression in mouse-bone-marrow-derived dendritic cells (BMDCs). Mouse-BMDCs were transduced with a MOI of 2 using the LVs expressing GFP under the indicated promoters. (**a**) Results of time-course flow cytometric analyses performed at Days 3 and 7 post-transduction, to compare MFIs among the different LVs. (**b**) MFI normalized by the percentage of transduced cells expressing GFP. Histograms show the means of two replicates. Error bars indicate S.E.M.

We then tested the same LVs on mouse BMDCs isolated from C57BL/6 mice. In contrast to HEK 293T cells, LVs expressing GFP under promoters PrDrive2, 6 and 8 had the highest transgene expression levels at Days 3 (Figure 2a) and 7 (Figure 2b) post-transduction. There was no significant difference in MFI between Days 3 and 7 post-transduction.

**Figure 3:**
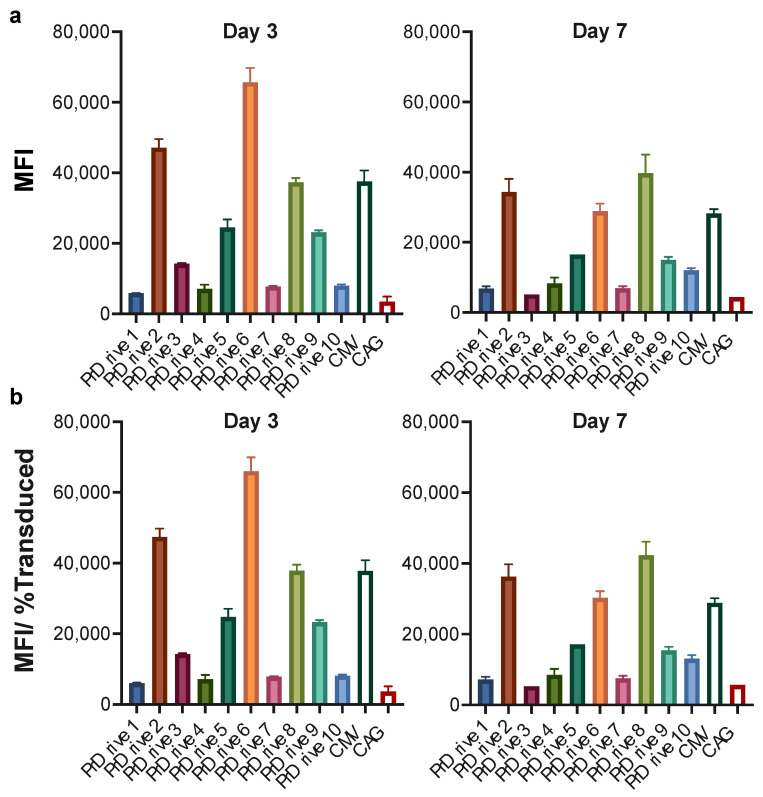
Impact of vector promoter on transgene expression in monkey-monocyte-derived dendritic cells. Monkey-monocyte-derived DCs were transduced with an MOI of 4 using the LVs expressing GFP under the indicated promoters. (**a**) Results of time-course flow cytometric analyses performed at the indicated time points to compare MFIs among the different LVs. (**b**) MFI normalized for the percentage of transduced cells expressing GFP. Histograms represent means of two replicates. Error bars indicate S.E.M.

We repeated these experiments on monkey- and human-monocyte-derived DCs (MDDCs) to determine whether species-specific differences exist in transgene expression levels driven by these promoters. Similar to what we observed in murine BMDCs, the LVs expressing GFP under the PrDrive2, 6, and 8 and CMV promoters had the highest transgene expression levels in monkey MDDCs at Days 3 and 7 (Figure 3a,b) post-transduction. These results suggest that the LVs expressing GFP under promoters PrDrive2, 6 and 8 and CMV are the most efficient at driving gene expression in both mouse and monkey DCs. 

**Figure 4:**
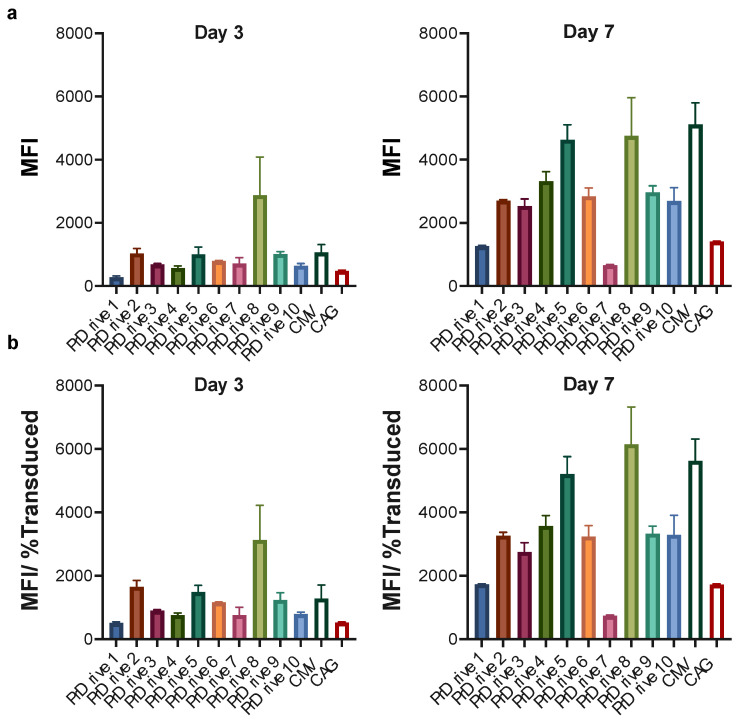
Impact of vector promoter on transgene expression in human-monocyte-derived dendritic cells (MDDCs). Human MDDCs were transduced with an MOI of 4 using the LVs expressing GFP under the indicated promoters. (**a**) Results of time-course flow cytometric analyses performed at the indicated time points to compare MFIs among the different LVs. (**b**) MFI normalized for the percentage of transduced cells expressing GFP. Histograms represent the means of two replicates. Error bars indicate S.E.M.

In human DCs, we observed higher transgene expression levels from the LV expressing GFP under the PrDrive8 promoter at Day 3 post-transduction; however, this advantage was lost at Day 7 post-transduction, where an increase in GFP expression was observed for all LVs (Figure 4a,b), with the LVs expressing GFP under the PrDrive5 and 8 and CMV promoters demonstrating the highest transgene expression levels (Figure 4b). 

**Figure 5:**
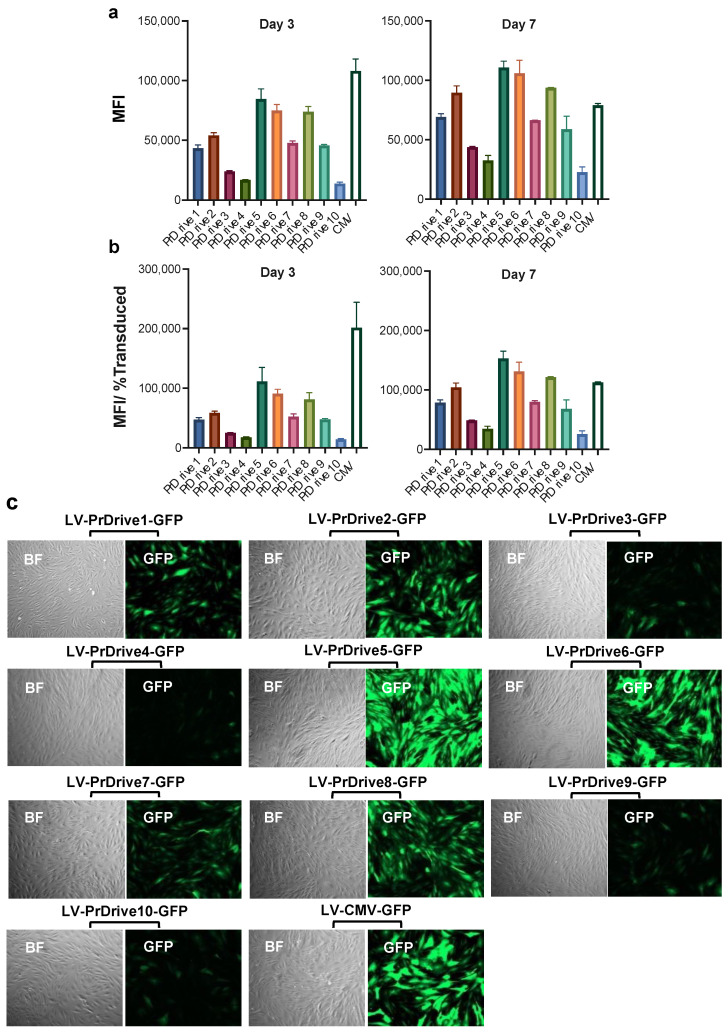
Impact of vector promoter on transgene expression in cynomolgus macaque skeletal muscle cells. Monkey skeletal muscle cells were transduced with an MOI of 0.5 using the LVs expressing GFP under the indicated promoters. (**a**) Results of time-course flow cytometric analyses performed at the indicated time points to compare mean fluorescence intensity (MFI) among the different LVs. (**b**) MFI normalized by the percentage of transduced cells expressing GFP. Histograms show the means of two replicates. Error bars indicate S.E.M. (**c**) Fluorescence microscopy images of monkey muscle cells transduced with the indicated LVs at 7 days post-transduction.

Finally, given the importance of muscle cells in the maintenance of an antigen reservoir following IDLV injection [16], we tested the LVs on monkey skeletal muscle cells. As shown in Figure 5a–c, LVs expressing GFP under promoters PrDrive2, 5, 6 and 8, together with the CMV promoter, had the highest transgene expression levels at Days 3 and 7 post-transduction. Interestingly, LVs expressing GFP under promoters PrDrive2, 5, 6 and 8 and CMV were the most efficient in both monkey muscle cells and DCs, implying that the same LVs could be used to achieve high transgene expression in both DCs and muscle cells in vivo.

### 3.2. Impact of Vector Promoter on the Magnitude of IDLV-Induced T Cell Responses

**Figure 6:**
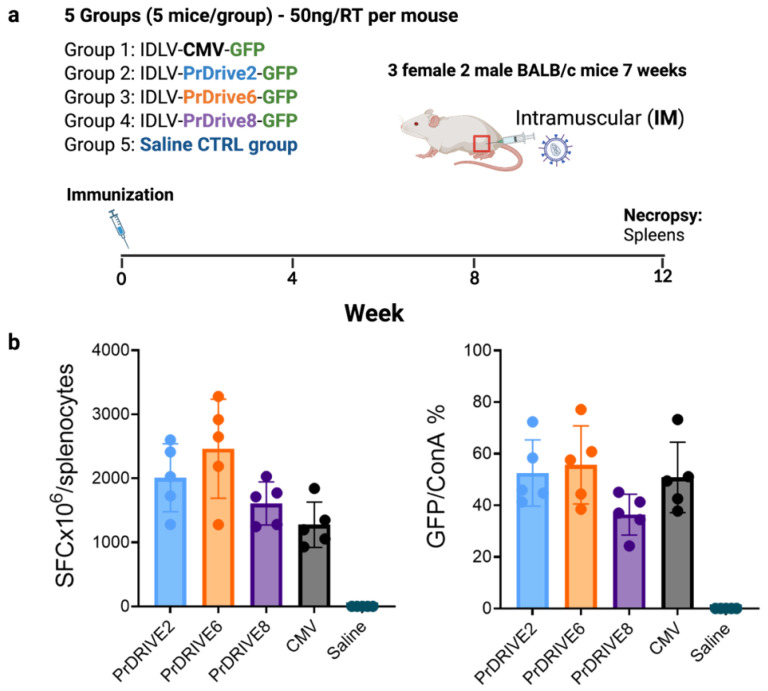
Magnitude of T cell responses in mice immunized with IDLVs expressing GFP under different promoters. (**a**) A total of 25 BALB/c mice were immunized intramuscularly with 50 ng RT/mouse corresponding to 5 × 10^6^ transducing units (TU) of the indicated IDLVs or saline. Spleens were harvested 12 weeks post-immunization to measure T cell responses. (**b**) Magnitudes of GFP-specific T cell responses induced by the indicated IDLVs at 12 weeks post-immunization, as measured by IFN-γ ELISpot. Data are expressed (left panel) as numbers of specific spot-forming cells (SFCs) per million cells and (right panel) as % of GFP-specific SFCs normalized per ConA-induced SFCs, to account for potential differences in T cell responsiveness to stimuli among samples. Background responses in unstimulated wells (medium only) were subtracted. GFP = green fluorescent protein; ConA = concavalin A.

To determine whether the higher transgene expression levels achieved in vitro by these LVs translated to a high immune response in vivo, we immunized five groups of five BALB/c mice. Each group of mice was immunized intramuscularly with the corresponding IDLV expressing GFP under promoters PrDrive2, 6 and 8 and CMV, or with a saline sham injection (Figure 6). T cell responses were measured by IFN-γ ELISpot using splenocytes collected 3 months after the single immunization. As shown in Figure 6b and Supplementary Figure S6, the IDLVs expressing GFP under the PrDrive6 promoter induced higher T cell responses than the other IDLVs, although differences were not statistically significant. These data suggest that in vivo, these novel IDLVs induce antigen-specific T cell responses of similar magnitude to those produced by the IDLV with expression driven by the commonly used CMV promoter.

## 4. Discussion

Lentiviral vectors (LVs) offer many advantages as gene delivery systems for in vitro, ex vivo and in vivo approaches. Their relatively flexible genome and ability to transduce dividing and nondividing cells, combined with their potential for cell-specific pseudotyping, provides a useful tool for several applications in both experimental and therapeutic settings [4]. In the clinic, lentiviral vector-based gene-engineering strategies have successfully been used for the chimeric antigen receptor (CAR)-T and CAR-NK cell therapy of cancer due to their high transduction efficiency and low risk of oncogenic insertion [18,19,20]. In addition to their use in the development of these cell-based immunotherapies, integrase defective lentiviral vectors (IDLVs) have shown promise in pre-clinical vaccine studies against pathogens such as HIV, influenza, and SARS-CoV-2 [12,13,21,22,23,24], and in clinical studies as a DC-targeting vaccines against metastatic sarcoma [25,26].

One advantage of IDLVs as vaccine platforms is their ability to efficiently transduce DCs [27,28]. Most DC-based vaccine strategies previously tested against infectious diseases and cancer have used ex vivo approaches, where DCs differentiated from donor-derived monocytes were pulsed with synthetic peptides in the presence of activating cytokines and then re-infused in the donor to activate antigen-specific cytotoxic T lymphocytes (CTLs) [29,30]. A limitation of these approaches is the short-term antigen presentation provided by peptide pulsing or exogenous antigen-loading strategies. One strategy to overcome these limitations is the use of an antigen delivery platform such as IDLV that can effectively transduce DCs in vivo [31] and provide a more durable antigenic presentation.

We have previously shown that SIV-based IDLVs have significantly higher transduction efficiency than the HIV-based IDLVs on both simian and human DCs [32]. Here, we generated and tested ten novel SIV-based lentiviral vector constructs expressing the antigen of interest under different hybrid promoters and assessed their impact on transgene expression levels in DCs as well as in muscle cells, given the importance of the latter in maintaining an antigen reservoir at the injection site [16].

We observed that SIV-based LVs expressing GFP under promoters PrDrive2, 6 and 8 showed significantly higher transgene expression levels in both murine and monkey DCs when compared to the other LVs. Interestingly, these same vectors also outperformed the other constructs in monkey muscle cells, with PrDrive8 being also the most efficient vector in human DCs. 

We demonstrated that these three novel IDLVs induce a high magnitude and durable antigen-specific T cell response when injected intramuscularly in mice. These results suggest that high transgene expression in DCs and muscle cells translates to high magnitude and durable immune responses in vivo.

In summary, we generated ten novel lentiviral vectors expressing the transgene of interest under different hybrid promoters containing different enhancer and 5′ UTR sequences. Our results suggest that the choice of the promoter to drive the expression of the transgene of interest plays an important role in the efficiency of transgene expression. These new lentiviral vectors can be used as LVs or IDLVs to achieve higher transgene expression levels in target cells for a variety of applications, including in vitro experiments and ex vivo or in vivo preventive and therapeutic approaches.

## Figures and Tables

**Table 1:**
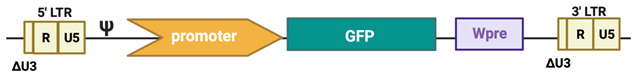
Characteristics of the ten hybrid promoters cloned into an SIV-based LV transfer vector plasmid [4] as shown in the diagram below (created with BioRender.com). Each of these promoters is composed of an enhancer, a core promoter and a 5′ UTR. The promoters were obtained from Invivogen. UTR = untranslated region; bp = base pair; Hcmv = human cytomegalovirus; mCMV = murine cytomegalovirus; hEF1 = human elongation factor 1; HTLV = human T-lymphotropic virus; mTyr = mouse tyrosinase; SV40 = simian virus 40; hFerL= human ferritin light chain; hAldA = human aldehyde dehydrogenase; chEF1 = chimpanzee elongation factor 1.

Promoter Name	Size	Enhancer	Core Promoter	5′ UTR
PrDrive1	929 bp	hCMV	hEF1	HTLV
PrDrive2	947 bp	mCMV	hEF1	HTLV
PrDrive3	759 bp	SV40	hEF1	HTLV
PrDrive4	723 bp	mTyr	hEF1	HTLV
PrDrive5	827 bp	hCMV	hCMV	HTLV
PrDrive6	657 bp	SV40	hCMV	HTLV
PrDrive7	1656 bp	hCMV	hFerL	chEF1
PrDrive8	1674 bp	mCMV	hFerL	chEF1
PrDrive9	1486 bp	SV40	hFerL	chEF1
PrDrive10	1530 bp	hAldA	hFerL	chEF1

## Data Availability

The data presented in this study are available on request from the corresponding author.

## Supplementary Materials

The following supporting information can be downloaded at: https://www.mdpi.com/article/10.3390/v15112255/s1, Figure S1. Gating strategy for 293T-Lenti-X; Figure S2. Gating strategy for Mouse Bone Marrow Derived DCs. Figure S3. Gating strategy for Monkey Monocytes Derived DCs. Figure S4. Gating strategy for Human Monocytes Derived DCs. Figure S5. Gating strategy for Monkey Skeletal Muscle cells (MK-6167). Figure S6. Magnitude of T-cell responses in mice immunized with IDLVs expressing GFP under different promoters.
