# Supplementary material for: Integrase Defective Lentiviral Vector Promoter Impacts Transgene Expression in Target Cells and Magnitude of Vector-Induced Immune Responses
Source: Viruses. 2023 Nov 14;15(11):2255. doi: 10.3390/v15112255 (PMC10674321; doi:10.3390/v15112255)
Supplement: Supplementary file 1 [file viruses-15-02255-s001.zip › viruses-2659860-supplementary.pdf]

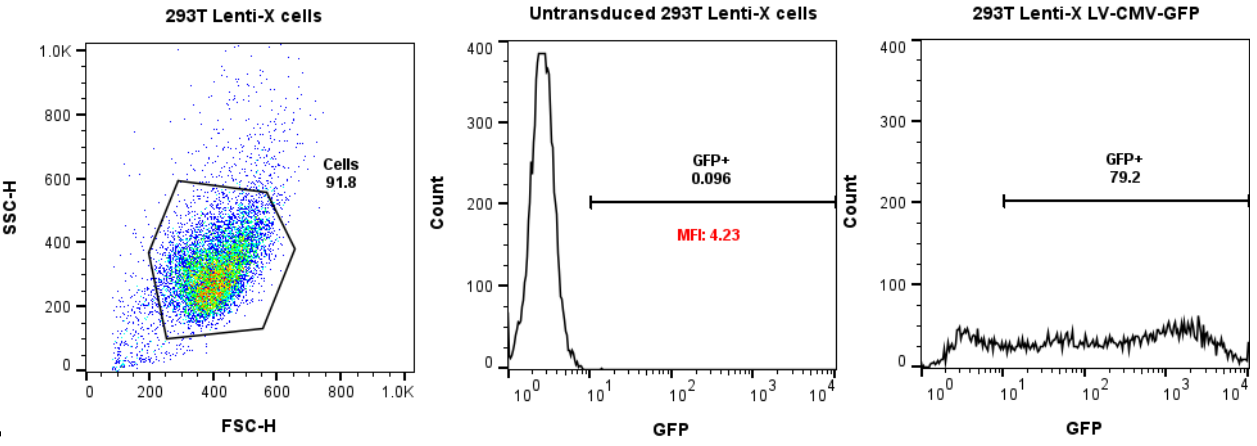

WS

Figure S1. Gating strategy for 293T-Lenti-X.

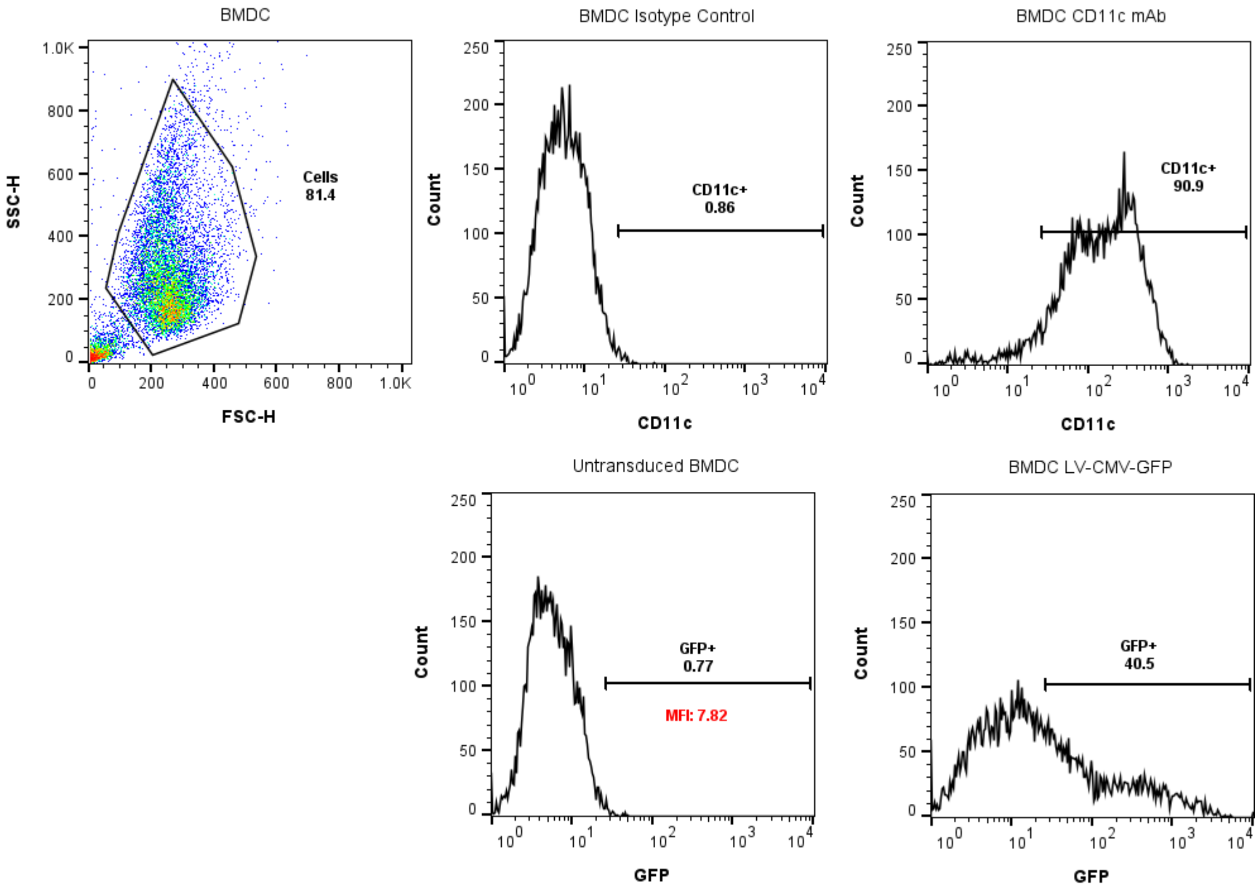

Figure S2. Gating strategy for Mouse Bone Marrow Derived DCs.

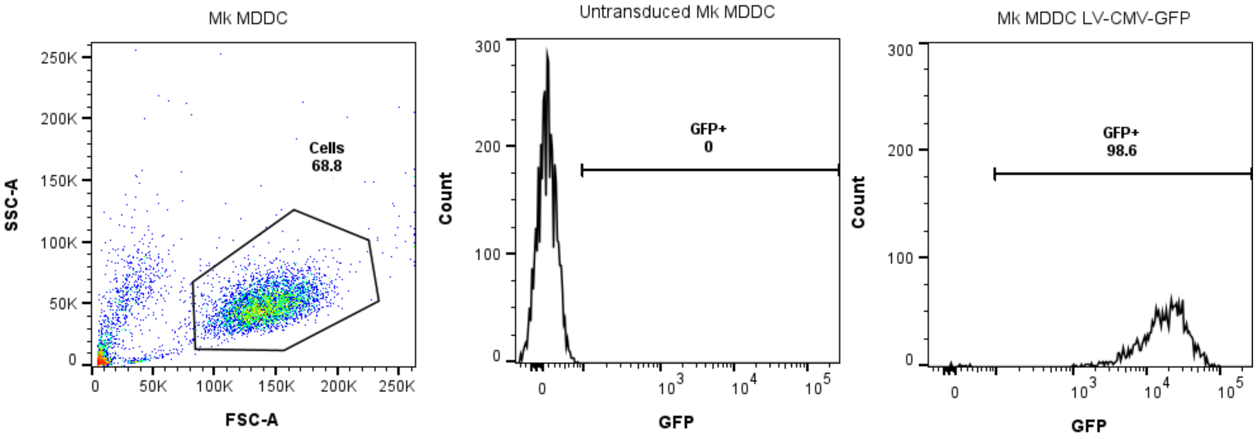

Figure S3. Gating strategy for Monkey Monocytes Derived DCs.

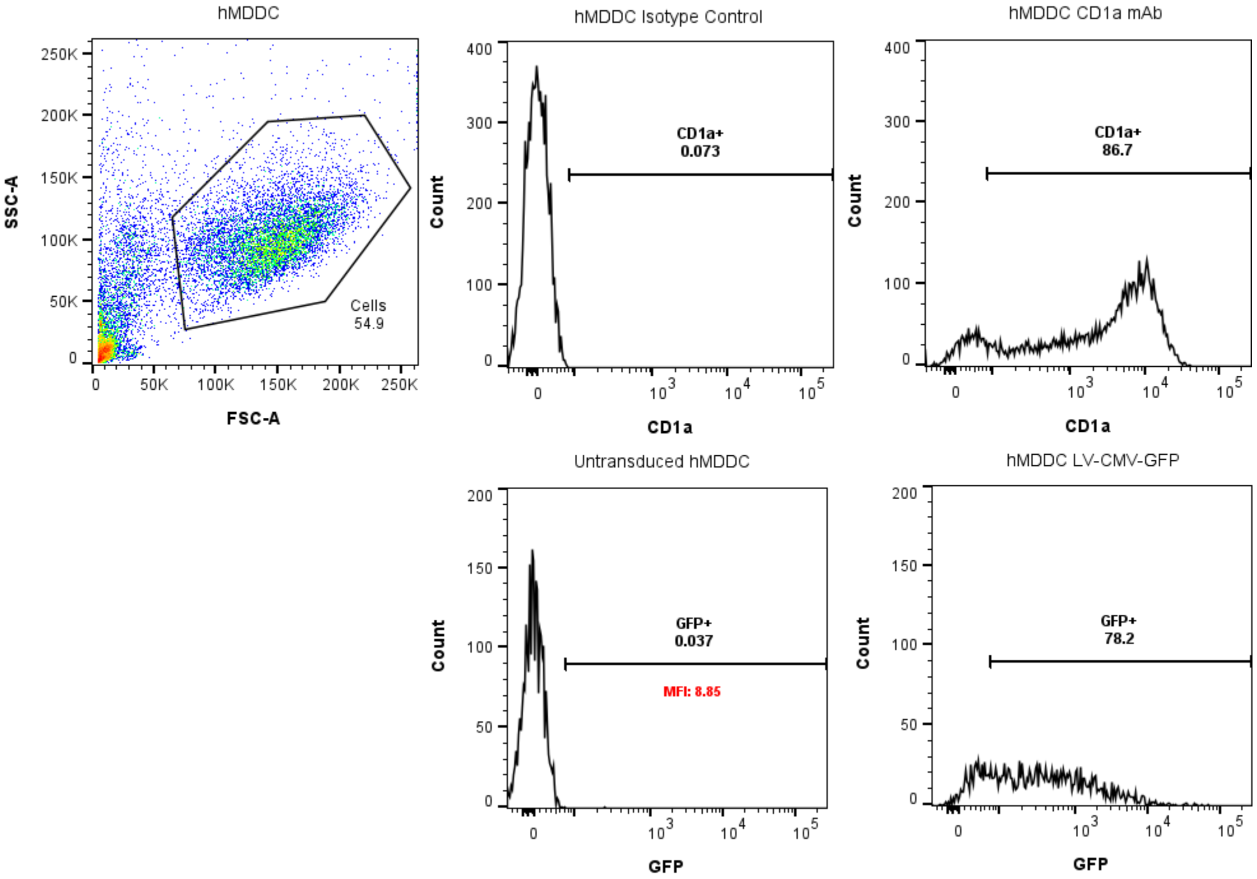

Figure S4. Gating strategy for Human Monocytes Derived DCs.

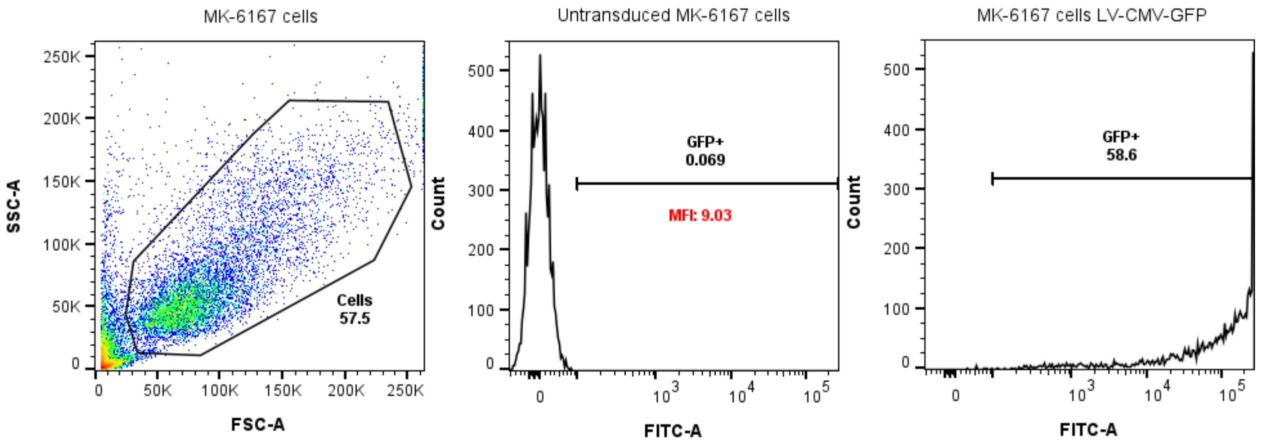

Figure S5. Gating strategy for Monkey Skeletal Muscle cells (MK-6167).

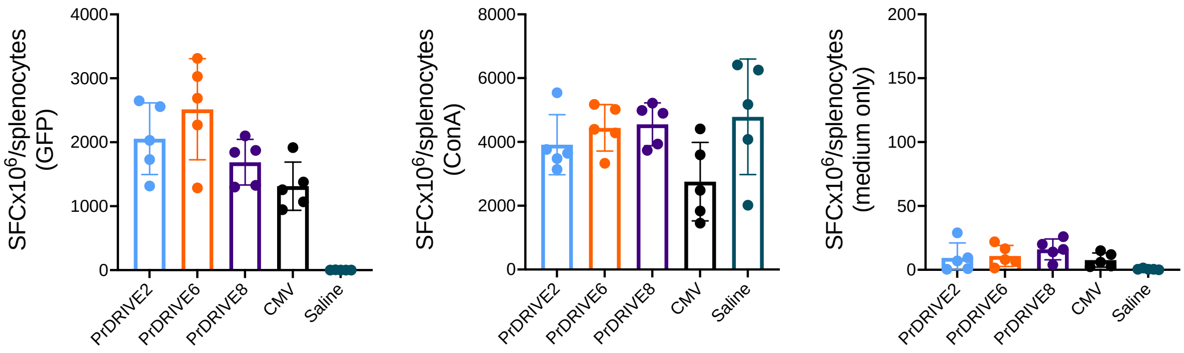

a b c

Figure S6. Magnitude of T-cell responses in mice immunized with IDLVs expressing GFP under different promoters. 25 BALB/c mice were immunized intramuscularly with 50 ng RT/mouse corresponding to 5x10^6 transducing units (TU) of the indicated IDLVs. Spleens were harvested 12 weeks post-immunization to measure T cell responses (**a**). Magnitude of GFP-specific T cell responses induced by the indicated IDLVs at 12 weeks post-immunization as measured by IFN-γ ELISpot. Data are expressed as numbers of GFP-specific (**a**) or ConA-induced (**b**) spot forming cells (SFCs) per million cells. Background responses in unstimulated wells (medium only) are shown in panel (**c**). A response was considered positive when there was at least a 2-fold increase in number of spots over medium-treated wells (background) with a minimum threshold of 50 SFCs per million splenocytes in the stimulated wells.
